# Physiological and pathological roles of the accommodation response in lower esophageal sphincter relaxation during wet swallows

**DOI:** 10.1038/s41598-021-87052-x

**Published:** 2021-04-12

**Authors:** Kazumasa Muta, Eikichi Ihara, Shohei Hamada, Hiroko Ikeda, Masafumi Wada, Yoshitaka Hata, Xiaopeng Bai, Yuichiro Nishihara, Yoshimasa Tanaka, Haruei Ogino, Yoshihiro Ogawa

**Affiliations:** 1 Department of Medicine and Bioregulatory Science, Graduate School of Medical Sciences, Kyushu University, 3-1-1 Maidashi, Higashi-ku, Fukuoka-shi, Fukuoka Japan; 2 Department of Gastroenterology and Metabolism, Graduate School of Medical Sciences, Kyushu University, 3-1-1 Maidashi, Higashi-ku, Fukuoka-shi, Fukuoka 812-8582 Japan

**Keywords:** Gastroenterology, Gastrointestinal diseases, Motility disorders, Achalasia, Physiology

## Abstract

The preparatory accommodation response of lower esophageal sphincter (LES) before swallowing is one of the mechanisms involved in LES relaxation during wet swallows, however, the physiological and/or pathological roles of LES accommodation remain to be determined in humans. To address this problem, we conducted a prospective observational study of 38 patients with normal high-resolution manometry (HRM) and 23 patients with idiopathic esophagogastric junction outflow obstruction (EGJOO) to assess dry and wet swallows. The LES accommodation measurement was proposed for practical use in evaluating the LES accommodation response. Although swallow-induced LES relaxation was observed in both dry and wet swallows, LES accommodation (6.4, 3.1–11.1 mmHg) was only observed in wet swallows. The extent of LES accommodation was impaired in idiopathic EGJOO (0.6, − 0.6–6 mmHg), and the LES accommodation measurement of patients with idiopathic EGJOO (36.8, 29.5–44.3 mmHg) was significantly higher in comparison to those with normal HRM (23.8, 18–28.6 mmHg). Successful LES relaxation in wet swallowing can be achieved by LES accommodation in combination with swallow-induced LES relaxation. Impaired LES accommodation is characteristic of idiopathic EGJOO. In addition to the IRP value, the LES accommodation measurement may be useful for evaluating the LES relaxation function in clinical practice.

## Introduction

Esophageal motility function is responsible for the esophageal phase of swallowing, and coordinated esophageal motility function that is exquisitely controlled by the neuromuscular response is indispensable for daily dietary intake^1^. While not eating, both the upper esophageal sphincter (UES) and lower esophageal sphincter (LES) maintain basal pressure to prevent the reflux of the gastric contents into the esophagus and pharynx. During eating it is necessary to achieve timely and sufficient LES relaxation accompanied by subsequent peristalsis of the esophageal body, to allow the passage of food into the stomach. In particular, such LES relaxation during eating plays a key role in the successful transportation of ingested food into the stomach. It is well known that LES relaxation is induced by the swallowing action itself, which can be termed as swallow-induced LES relaxation. In addition to this response, LES relaxation is also caused by the physical stimulation of oral cavity and/or pharynx by liquid and food before the swallowing action, which is termed pharyngeal water stimulation (PWS)-induced LES relaxation^2^. Since this PWS-induced LES relaxation appears before the swallowing action, it can be considered the LES’s accommodation of the preparatory response for swallow-induced LES relaxation.

This concept of LES accommodation reminds us of gastric accommodation with relaxation of the proximal stomach. The gastric accommodation response plays an important role in the gastric motility function in order to reserve ingested food for a certain period of time, and it is caused by two distinct mechanisms, including gastric receptive relaxation and gastric adaptive relaxation^3^. Gastric receptive relaxation is caused by the physical stimulation of pharynx while gastric adaptive relaxation is induced by contact and stretch stimulation of the gastric fundus directly, by ingested food. From the viewpoint of the relaxation mechanisms, PWS-induced LES relaxation in the esophagus corresponds to gastric receptive relaxation. It could therefore be referred to as receptive LES relaxation. It is reasonable to consider that LES has a similar accommodation response to the proximal stomach since LES is located anatomically next to the gastric proximal stomach. In addition, the smooth muscles of both the LES and proximal stomach are characterized by tonic contractility where the smooth muscles maintain a certain basal tone at rest and relax upon stimulation of ingested food during eating. However, it remains to be determined how the LES accommodation response, including receptive LES relaxation, contributes to successful LES relaxation during eating.

Major dysfunction of esophageal motility is collectively referred to as esophageal motility disorder (EMD). A representative example of EMD is achalasia, which reduces quality of life, work productivity and limits oral intake due to difficulty in swallowing, and can also lead to life-threatening diseases, such as aspiration pneumonia^4^. A novel disease concept termed esophagogastric junction outflow obstruction (EGJOO) has been established based on high-resolution manometry (HRM) together with Chicago classification (CC) ver3.0^5^. While achalasia is characterized by impaired relaxation of the LES as well as impaired esophageal body peristalsis, EGJOO is defined as a disorder of impaired LES relaxation with intact esophageal body peristalsis. It is important to elucidate the pathophysiology of EGJOO, since it is possible that EGJOO could be a precursor of achalasia, and that treatment for EGJOO may prevent the onset of achalasia. Although we recently indicated in a pilot study that EGJOO might be characterized by impairment of receptive LES relaxation^6^, further investigation is required.

We aimed to elucidate the mechanisms of physiological LES relaxation during dry and wet swallowing, by assessing how LES accommodation (represented by receptive LES relaxation), together with swallow-induced LES relaxation, contribute to timely and sufficient LES relaxation, and to determine the mechanisms of the impaired LES relaxation in EGJOO, by focusing on the pathological role of the LES accommodation response.

## Results

### The clinical characteristics of the enrolled patients

The clinical characteristics of the patients with normal HRM, patients with EGJOO and patients with achalasia are shown in Table 1. There were no significant differences in the demographic data among of the three groups. The Eckardt score of the patients with achalasia was significantly higher than that of the patients with normal HRM. The dysphagia item of the Eckardt score obtained in EGJOO patients tended to be higher, and that obtained in achalasia patients was significantly higher in comparison to the scores of patients with normal HRM. In addition, the regurgitation item of the Eckardt score obtained in achalasia patients was significantly higher than that obtained in EGJOO patients.

**Table 1:** Clinical characteristics of the patients with normal HRM and patients with EGJOO and Achalasia.

Clinical characteristics	Normal HRM (n = 38)	EJGOO (n = 23)	Achalasia (n = 33)
Age, years	60.5 (48.5–68.0)	66.0 (57.0–68.0)	65 (37–72.5)
Sex, female/male	21/17	6/17	18/31
Height, cm	160 (157.0–163.8)	156.8 (150.5–162.0)	163 (153–171.5)
Body weight, kg	54.6 (48.6.–61.6)	51 (46.4–60.5)	58 (46–65.8)
Body mass index, kg/m^2^	20.6 (18.6–24.2)	20.9 (18.7–22.8)	21.4 (19.2–23.6)
**Eckard score**			
Total	2.1 ± 0.43	3.0 ± 0.57	4.2 ± 0.42^a^
Weight loss	0.59 ± 0.18	0.83 ± 0.22	0.94 ± 0.16
Dysphagia	0.37 ± 0.09	0.83 ± 0.25	1.5 ± 0.19^a^
Retrosternal pain	0.56 ± 0.15	0.78 ± 0.24	0.56 ± 0.13
Regurgitation	0.59 ± 0.13	0.56 ± 0.18	1.2 ± 0.15^b^

*EGJOO* esophagogastric junction outflow obstruction; *HRM* high-resolution manometry.

Results are shown as the median (interquartile range) or mean ± standard error.

^a^*p* < 0.05 vs. Normal HRM.

^b^*p* < 0.05 vs. EGJOO.

### Difference in HRM parameters between dry and wet swallows in patients with normal HRM

**Figure 1:**
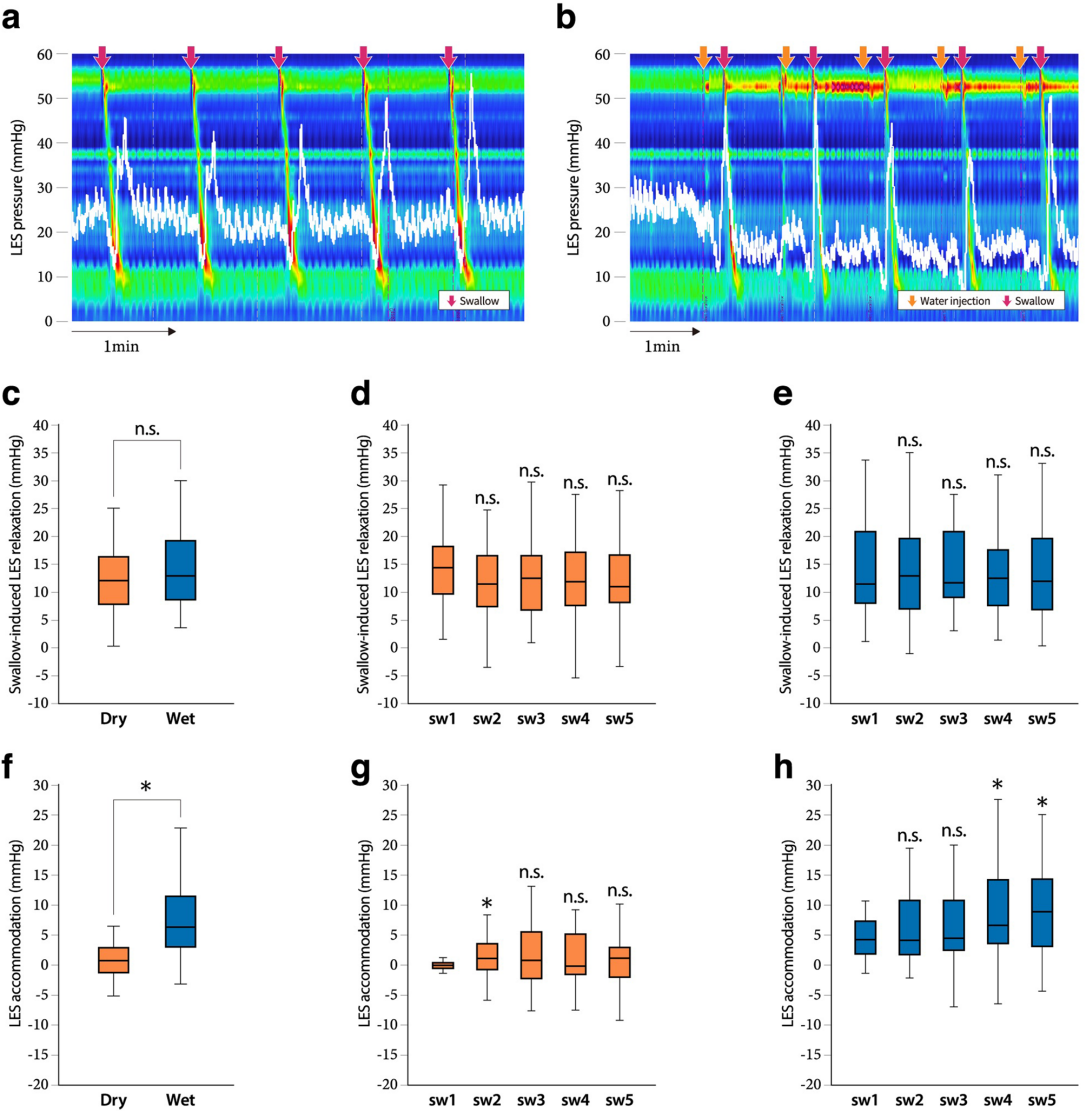
A comparison of the HRM findings between dry and wet swallows for swallow-induced LES relaxation and LES accommodation. a, b, A series of representative recordings of dry (**a**) and wet (**b**) swallows. Red arrows indicate the swallowing action while orange arrows indicate the timing of water injection into the oral cavity. The patients were asked to hold 5 mL water in the pharynx for 5 s before swallowing. (**c**–**e**), Cumulative data of swallow-induced LES relaxation in a total of five dry and wet swallows (**c**) and in each dry (**d**) and wet (**e**) swallow from the 1st to 5th swallows. (**f**–**h**), Cumulative data on LES accommodation in a total of five dry and wet swallows (**f**) and in each dry (**g**) and wet (**h**) swallow from the 1st to 5th swallows. Data are shown as the median (interquartile range). A paired t-test was conducted (**c** and **f**). *A statistically significant difference between the two groups. n.s., no significant difference between the two groups. A repeated measure analysis of variance followed by Dunnett’s test was conducted (**d**, **e**, **g**, **h**), with the value of the 1st swallow (sw1) set as the control. *A statistically significant difference vs. sw1 (*p* < 0.05). n.s., no significant difference vs. sw1.

A series of representative recordings of dry and wet swallows is shown in Fig. 1a,b. In dry swallows, the LES pressure transiently decreased immediately after the swallowing action and recovered to the BLESP after esophageal body peristalsis. In contrast, in wet swallows, the LES pressure decreased simply from the administration of 5 mL of water into the oral cavity; this was particularly obvious at the 1st wet swallow. The LES pressure further transiently decreased by the action of wet swallowing. The LES pressure did not recover to the BLESP after esophageal body peristalsis.

The basal HRM parameters in dry and wet swallows (n = 38) are shown in Table 2. Regarding the LES function, the mean IRP in dry swallowing (17.9, 13.4–21.3 mmHg) was significantly higher than that in wet swallowing (10.9, 9.8–13.0 mmHg). There were no significant differences in the BLESP (29.6, 25.6–36.4 vs. 31.8, 23.8–36.4 mmHg), IBP at LES relaxation (6.3, 3.2–11.3 vs. 5.6, 4.8–8.3 mmHg) or IBP average max (13.9, 10.8–21.5 vs. 12.3, 10.8–15.7 mmHg) between dry and wet swallows. Regarding the esophageal body function, the mean values of DCI (1006, 533.8–1983.3 mmHg·cm·s) in dry swallowing was significantly lower than that in wet swallowing (1765.1, 904.2–2243.9 mmHg·cm·s). The mean value of DL (5.9, 4.8–6.7 s) in dry swallowing was also significantly shorter than that in wet swallowing (7.2, 6.5–7.6 s).

**Table 2:** HRM pressure topography parameters of dry and wet swallows in the patients with normal HRM and of wet swallow in the patients with EGJOO and patients with achalasia.

	Normal HRM (n = 38)		EGJOO (n = 23)	Achalasia (n = 33)
	Dry swallow	Wet swallow	Wet swallow	Wet swallow
*Conventional HRM parameters*				
**LES function, mmHg**				
BLESP	29.6 (25.6–36.4)	31.8 (23.8–36.4)	41.2 (33.8–50.6)^b^	33.2 (24.1–42.5)
4 s-IRP	17.9 (13.4–21.3)	10.9 (9.8–13.0)^a^	20.5 (18.3–23.6)^b^	22.1 (16.6–29.7)^b^
IBP at LESR	6.3 (3.2–11.3)	5.6 (4.8–8.3)	8.7 (5.2–12.7)^b^	n.a
IBP average max	13.9 (10.8–21.5)	12.3 (10.8–15.7)	17.1 (12.5–25.2)^b^	n.a
**Esophageal body contractility**				
DCI, mm Hg·cm·s	1006 (533.8–1983.3)	1765.1 (904.2–2243.9)^a^	2049.6 (1366.2–2927.1)	n.a
DL, s	5.9 (4.8–6.7)	7.2 (6.5–7.6)^a^	6.1 (5.4–6.6)^b^	n.a
**Original HRM parameters**				
Swallow-induced LES relaxation, mmHg	12.1 (8.0–16.1)	12.8 (8.6–19.1)	14.2 (11.4–19.7)	6.1(3.4–7.8)^b,c^
LES accommodation, mmHg	0.7 (− 1.1–2.8)	6.4 (3.1–11.1)^a^	0.6 (− 0.6–6.0)^b^	1.5 (− 3.1–6.9)^b^
Receptive LES relaxation, mmHg	n.a	2.6 (0.2–4.1)	− 1.2 (− 3.0–0.9)^b^	n.a
LES accommodation measurement, mmHg	n.a	23.8 (18.0–28.6)	36.8 (29.5–44.3)^b^	32.6 (23.6–39.5)^b^

*EGJOO* esophagogastric junction outflow obstruction; *HRM* high-resolution manometry; *LES* lower esophageal sphincter; *IRP* integrated relaxation pressure; *IBP* intrabolus pressure; *LESR* lower esophageal sphincter relaxation; *DCI* distal contractile integral; *DL* distal latency.

Results are shown as the median (interquartile range).

^a^*p* < 0.05 vs. Dry swallow in normal HRM.

^b^*p* < 0.05 vs. Wet swallow in normal HRM.

^c^*p* < 0.05 vs. EGJOO.

### Roles of swallow-induced LES relaxation and LES accommodation in dry and wet swallows in patients with normal HRM

We examined how swallow-induced LES relaxation contributed to the LES relaxation induced by dry and wet swallows. There was no significant difference in the extent of swallow-induced LES relaxation between dry (12.1, 8–16.1 mmHg) and wet (12.8, 8.6–19.1 mmHg) swallows in a total of 5 dry and wet swallows (Fig. 1c, Table 2). In a sub-analysis focusing on each swallow, there were also no significant difference in the extent of swallow-induced LES relaxation among 5 consecutive swallows both in dry and wet swallows (Figs. 1d,e).

**Figure 2:**
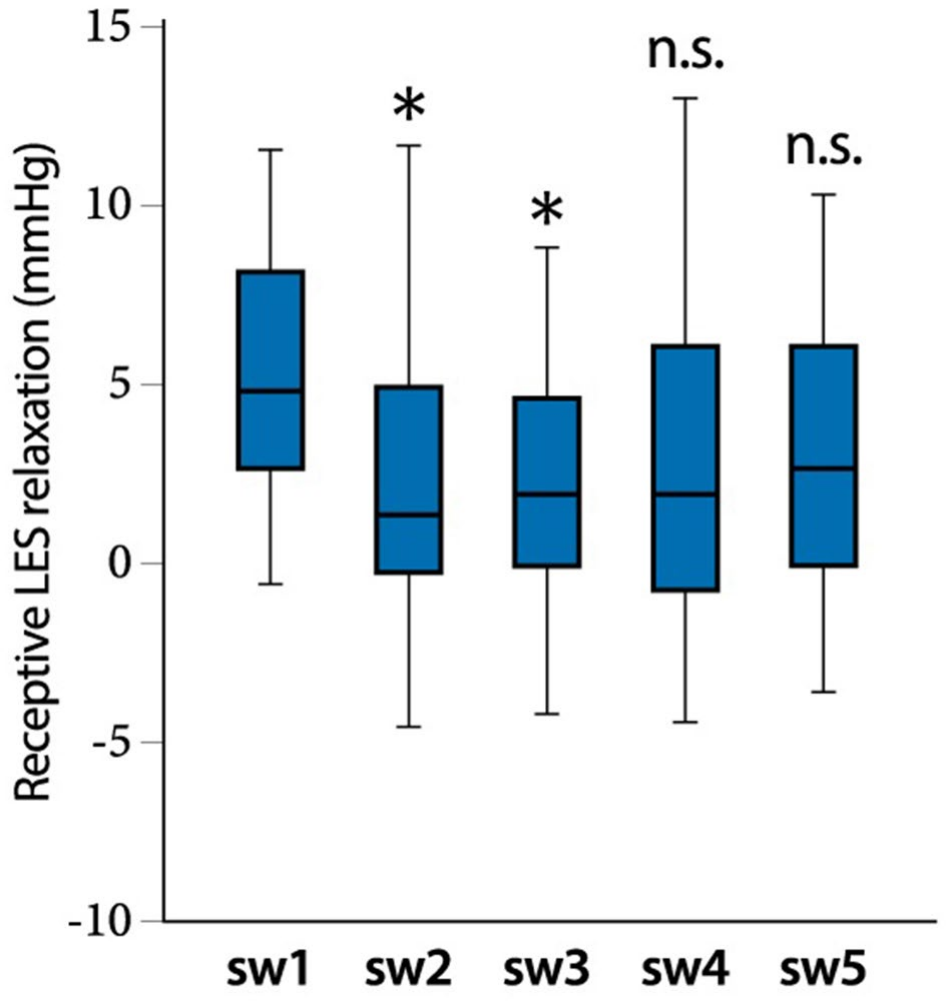
Receptive LES relaxation in wet swallowing. Cumulative data of receptive LES relaxation in each wet swallow from the 1st to 5th swallows. A repeated measure analysis of variance followed by Dunnett’s test was conducted, with the value of the 1st swallow (sw1) set as the control. *A statistically significant difference vs. sw1 (*p* < 0.05). n.s., no significant difference vs. sw1.

In contrast, no response in LES accommodation was seen in dry swallowing; the extent of LES accommodation observed in a total of 5 dry swallows was 0.7 mmHg (− 1.1–2.8, n = 38) (Fig. 1f, Table 2), while LES accommodation was observed in wet swallowing. The mean LES accommodation value in a total of 5 wet swallows was 6.4 mmHg (3.1–11.1, n = 23) (Fig. 1f). In a sub-analysis of LES accommodation focusing on each swallow, the extent of LES accommodation in wet swallowing showed a gradual but significant increase as wet swallowing was repeated (Fig. 1h) in comparison with dry swallowing (Fig. 1g). The extents of LES accommodation at the 4th (6.7, 3.6–14.1 mmHg) and 5th (8.9, 3.5–13.6 mmHg) wet swallows were significantly higher than that at the 1st swallow (4.2, 2.0–7.2 mmHg). Next, we examined the extent to which receptive LES relaxation contributed to LES accommodation in wet swallowing. receptive LES relaxation was mostly observed at the 1st swallow. The extents of receptive LES relaxation at the 2nd (0.6, − 1.0–3.9 mmHg) and 3rd (1.1, − 0.7–3.7 mmHg) wet swallows were significantly lower than that at the 1st swallow (4.2 mmHg, 2.0–7.1) (Fig. 2). Once the LES pressure decreased with the 1st receptive LES relaxation, the relaxant response seemed to be maintained in subsequent wet swallows. This continuous relaxant response could be considered part of LES accommodation.

### Assessment of LES relaxation during wet swallows in patients with EGJOO and patients with achalasia

**Figure 3:**
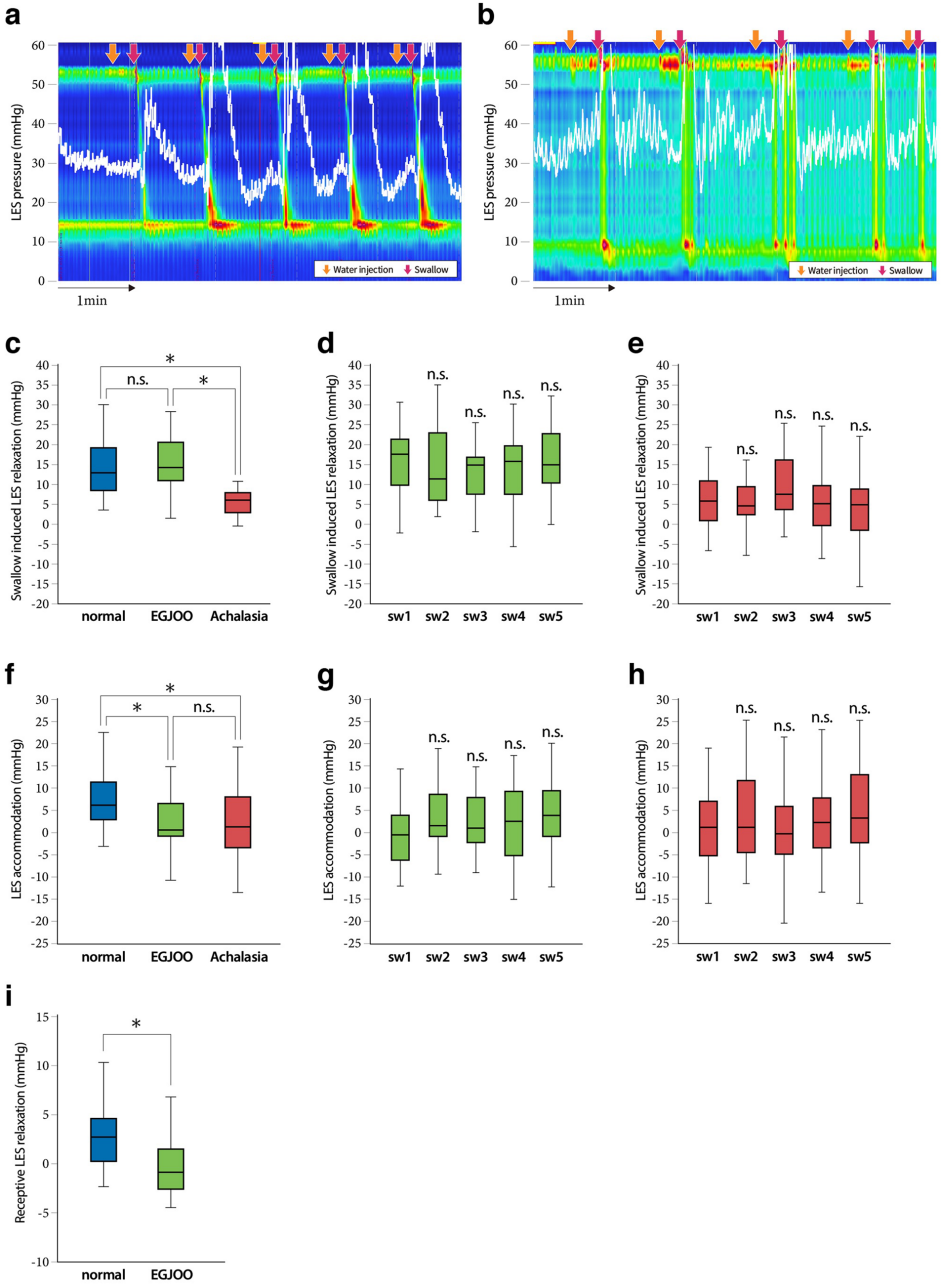
HRM findings of swallow-induced LES relaxation, and LES accommodation in EGJOO and achalasia. (**a**, **b**) The series of representative HRM recordings in a patient with EGJOO (**a**) and a patient with achalasia (**b**). Red arrows indicate the swallowing action, while orange arrows indicate the action of injecting water into the oral cavity. (**c**,**d**) Cumulative data on swallow-induced LES relaxation in a total of five swallows in a patient with EGJOO and a patient with achalasia (**c**) and in each swallow in a patient with EGJOO (**d**) and a patient with achalasia (**e**), from the 1st to 5th wallows. (**f**–**h**) Cumulative data on LES accommodation in a total of five swallows in a patient with EGJOO and a patient with achalasia (**f**) and in each swallow in a patient with EGJOO (**g**) and a patient with achalasia (**h**), from the 1st to 5th swallows. (**i**) Cumulative data of receptive LES relaxation in a total of five swallows in a patient with EGJOO. Data are shown as the median (interquartile range). A one-way ANOVA followed by Turkey’s multiple comparison test was conducted (**c** and **f**) and an unpaired t-test was conducted (**i**). *A statistically significant difference between the two indicated groups. n.s., no significant difference between the two indicated groups. A repeated measure analysis of variance followed by Dunnett’s test was conducted (**d**, **e**, **g**, **h**), with the value of the 1st swallow (sw1) set as the control. n.s., no significant difference vs. sw1.

A series of representative recordings of wet swallows in patients with EGJOO and patients with achalasia are shown in Fig. 3a,b. In patients with EGJOO, no apparent relaxation was induced by the administration of 5 mL of water into the oral cavity; however, the LES pressure transiently decreased with the wet swallowing action. After the 5th wet swallow, the LES pressure tended to be lower than the BLESP obtained before starting the 1st wet swallow. In contrast, no apparent decrease in LES pressure was observed in patients with achalasia. Because of the diagnostic criterion, the median IRP of patients with EGJOO (20.5, 18.3–25.6 mmHg) and patients with achalasia (22.1, 16.6–29.7 mmHg) was significantly higher than the upper limit of normal (15 mmHg). There were no significant differences in the median BLESP among three groups. Although they were not applicable for achalasia, there were significant differences in the IBP at LES relaxation (8.7, 5.2–12.6 vs. 5.6, 4.8–8.3 mmHg) and the IBP average max (17.1, 12.5–25.2 vs. 12.3, 10.8–15.7 mmHg) between the patients with EGJOO and the patients with normal HRM. In contrast, there were no significant differences in DCI (2049.6, 1366.2–2927.1 vs. 1765.1, 904.2–2243.9 mmHg·cm·s), or DL (6.1, 5.4–6.6 vs. 7.2, 6.5–7.6 s) between the patients with EGJOO and the patients with normal HRM.

Next we examined the impairment of the LES relaxation induced by wet swallowing in the patients with EGJOO and patients with achalasia. There was no significant difference in the swallow-induced LES relaxation between the patients with EGJOO (14.2, 11.4–19.7 mmHg) and the patients with normal HRM (12.8, 8.6–19.1 mmHg). However, the extent of swallow-induced relaxation among patients with achalasia (6.1, 3.4–7.8 mmHg) was significantly lower than that among patients with normal HRM and patients with EGJOO (Fig. 3c, Table 2). In addition, there were no significant differences in the extent of swallow-induced LES relaxation among the five wet swallows in patients with EGJOO and patients with achalasia (Fig. 3d,e). While a slight LES accommodation response was present, the median LES accommodation value in the patients with EGJOO (0.6, − 0.6–6 mmHg) was significantly lower than that of the patients with normal HRM (6.4, 3.1–11.1 mmHg) (Fig. 3f, Table 2). In addition, the extent of LES accommodation among patients with achalasia (1.5, − 3.1–6.9 mmHg) was also significantly lower than among patients with normal HRM but was similar to that among patients with EGJOO.

The LES accommodation response was not observed at the 1st wet swallow but did appear at the 4th and 5th wet swallows in patients with EGJOO (Fig. 3a,g), while it was completely abolished in patients with achalasia (Fig. 3b,h). In contrast, the receptive LES relaxation response was not seen in patients with EGJOO (− 1.2, − 3–0.9 mmHg), while it could not be technically assessed in patients with achalasia as they could not hold 5 mL water in the pharynx for 5 s (Fig. 3i, Table 2).

**Figure 4:**
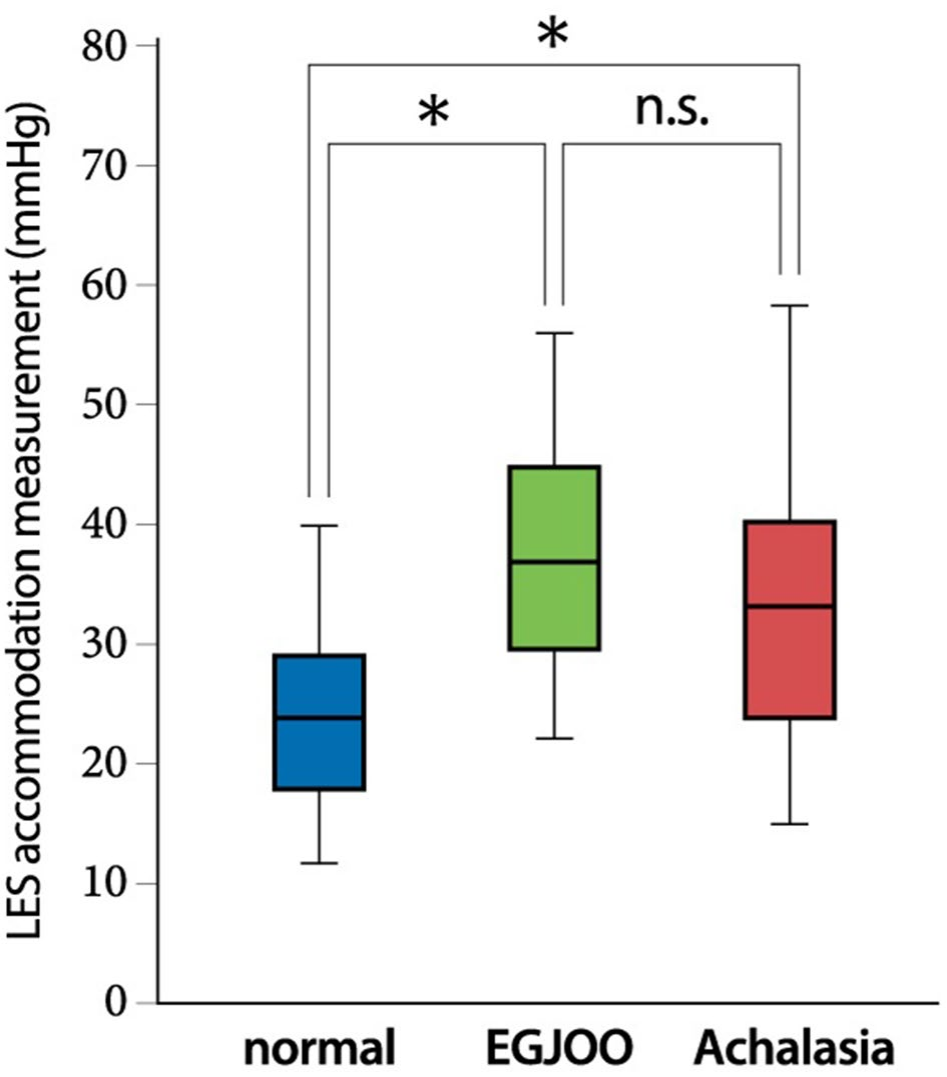
The LES accommodation measurement of the patients with normal HRM, patients with EGJOO and patients with achalasia. A one-way ANOVA followed by Turkey’s multiple comparison test was conducted. *A statistically significant difference between the indicated two groups. n.s., no significant difference between the two indicated groups.

Next, we examined whether or not the LES accommodation measurement was appropriate for evaluating the LES accommodation response. The LES accommodation measurements of patients with EGJOO (36.8, 29.5–44.3 mmHg) and patients with achalasia (32.6, 23.6–39.5 mmHg) were significantly higher than that of those with normal HRM (23.8, 18–28.6 mmHg) (Fig. 4, Table 2). One important issue in clinical practice is to differentiate EGJOO and normal HRM. As determined by an ROC analysis for the measurement of LES accommodation, the area under the curve (AUC) was 0.86. EGJOO can be discriminated from normal HRM using a cut-off value of 28.5 mmHg with 91.3% sensitivity and 73.7% specificity.

## Discussion

Timely and sufficient LES relaxation is indispensable for maintaining the esophageal motility function responsible for the esophageal phase of swallowing. Swallow-induced LES relaxation induced by the swallowing action itself plays a main role in LES relaxation during meal intake^1^. In addition to swallow-induced LES relaxation, however, another mechanism of LES relaxation called PWS-induced LES relaxation was proposed, which was induced by physical stimulation of the oral cavity and/or pharynx by liquid and food before the swallowing action^2^. We have focused on the mechanism of PWS-induced LES relaxation, which is similar to that of gastric receptive relaxation^7^. Thus, PWS-induced LES relaxation can be described as “receptive LES relaxation,” which was the term used in the present study. Although it has been reported that PWS-induced LES relaxation is associated with the onset of transient lower esophageal sphincter relaxation (TLESR)^8,9^, and with the inhibitory effects of esophageal peristalsis^7,10,11^, the physiological significance of this response remains to be fully determined. This was why we set the primary objectives of the present study.

In order to accomplish our objectives, we focused on comparing the LES relaxation of wet swallowing with that of dry swallowing, where no receptive LES relaxation was expected since a dry swallow does not stimulate the oral cavity and/or pharynx with liquid. As a result, we identified novel physiological responses related to wet swallowing. Receptive LES relaxation was observed especially in the 1st wet swallow, however it was hardly seen in the 2nd to 5th wet swallows (Fig. 1d). In contrast, there were no significant difference in the extent of swallow-induced LES relaxation, or in the level of IRP among the 1st to 5th wet swallows, indicating that a mechanism other than receptive LES relaxation was involved in LES relaxation during wet swallowing. Although the underlying mechanism is yet to be determined, before starting the protocol, we evaluated the decline in the LES pressure from the BLESP just before the action of wet swallowing , which was defined as the LES accommodation response in the present study. As a result, the LES accommodation response gradually became larger as wet swallowing repeated, the extent of LES accommodation at the 4th and 5th wet swallows was significantly greater in comparison to the 1st wet swallow (Fig. 1c). In other words, the level of LES pressure before the injection of liquid into the oral cavity gradually declined as wet swallows were repeated. It seemed that the response of receptive LES relaxation from the 2nd to the 5th wet swallows was scarcely observed since the LES pressure had decreased when liquid entered the oral cavity and pharynx. This means that both receptive and, so called, non-receptive LES relaxation contributed to the LES accommodation response where receptive LES relaxation mostly acted at the 1st wet swallow and non-receptive LES relaxation acted at the subsequent wet swallows. Since we will assume that non-receptive LES relaxation is caused in a similar manner to gastric adaptive relaxation, it will possibly be termed as adaptive LES relaxation ^3^. On the other hand, there was no LES accommodation, including receptive LES relaxation in dry swallowing (Fig. 1b). Interestingly, the extent of swallow-induced LES relaxation in dry swallowing was as large as that in wet swallowing (Fig. 1c). However, full LES relaxation could not be achieved in dry swallowing, indicating that swallowing-induced LES relaxation in combination with LES accommodation is necessary for successful LES relaxation during wet swallowing.

In the present study, we determined not only the physiological role of the LES accommodation response but also its pathological role in idiopathic EGJOO and achalasia. Patients with idiopathic EGJOO complain of various of symptoms similar to those of patients with achalasia, including chest pain, heartburn and choking on food. The diagnosis of EGJOO by HRM includes not only idiopathic EGJOO but also secondary EGJOO with a mechanical process including neoplastic lesions at the EGJ, eosinophilic esophagitis, rings and esophageal edema^12–16^. Other modalities, including EGD, esophagography and CT are additionally required to exclude secondary EGJOO. Although little is known about the etiology or pathogenesis of idiopathic EGJOO, we found that altered LES accommodation, especially altered receptive LES relaxation, is an underlying cause of idiopathic EGJOO. Both EGJOO and achalasia are disorders of EGJ outflow obstruction^5^. EGJOO is characterized by impaired LES accommodation but intact swallow-induced LES relaxation, whereas achalasia is characterized by both impaired LES accommodation and impaired swallow-induced LES relaxation. It is reasonable to consider that idiopathic EGJOO could represent a variant of achalasia or its early stage^17,18^. As for the treatment of idiopathic EGJOO, there is no established medical treatment available. Invasive symptomatic treatments, including balloon dilatation, botulinum injection, and more recently, per-oral endoscopic myotomy (POEM), which was originally developed as an endoscopic treatment for achalasia, have been applied to the idiopathic EGJOO patients with severe symptoms^13,19–21^. Considering that idiopathic EGJOO would be a disease condition in a reversible phase towards achalasia, it is important to generate the medical treatment of idiopathic EGJOO since this may also prevent the onset of achalasia. Thus, the impaired LES accommodation response identified in the present study could be a target for generation of the treatment for idiopathic EGJOO. Under such conditions, we have found that acotiamide, a newly developed prokinetic agent, currently being used in clinical practice for the treatment of functional dyspepsia in Japan, has significant potential in the treatment of idiopathic EGJOO, which is mediated by the normalization of impaired LES relaxation possibly through the improvement of LES accommodation^6^. It is considered that acotiamide increases the acetylcholine concentration at the neuromuscular junctions and enteric nerve through its actions as an inhibitor of acetylcholine esterase activity and via its enhancement of acetylcholine release by antagonizing the M_1_ and M_2_ muscarinic receptors in the enteric nerves^22,23^. It has been reported that acotiamide restored impaired gastric accommodation in patients with functional dyspepsia^22^. A similar mechanism might be involved in the restoration of both the impaired LES and gastric accommodation.

One of the important findings of the present study that will impact on its clinical significance is that it suggests a possible approach to the evaluation of LES relaxation. Based on CC ver3.0, the IRP is a gold standard for determining whether LES relaxation was impaired when IRP > the upper limit of normal is a diagnostic criterion for EGJ outflow obstruction disorders, including EGJOO and achalasia. However, we sometimes encounter cases in which it is difficult to judge whether the LES relaxation is normal or not when the IRP is close to upper limit of normal. Another approach (aside from IRP) has been awaited to evaluate the LES relaxation in clinical practice, and the assessment of LES accommodation and swallow-induced LES relaxation is a promising approach for determining whether the LES relaxation is normal or impaired. In this context, considering the practical use of evaluating the LES accommodation response, we have proposed the LES accommodation measurement. As a result, the LES accommodation measurement can be used to discriminate idiopathic EGJOO from a normal HRM study.

The present study is associated with some limitations. First, this prospective observational study was performed in a single center. Second, although the normal patients enrolled in the present study had completely normal HRM findings, they had some thoracoabdominal symptoms, which could be attributed to functional gastrointestinal disorders at the time of enrollment in this study. Completely healthy subjects could not be enrolled due to ethical issues. Since those symptoms proved to be independent of EMDs, setting them as normal patients might be acceptable in the present study.

In conclusion, timely and sufficient LES relaxation in wet swallowing can be achieved by LES accommodation in combination with swallow-induced LES relaxation. In addition to receptive LES relaxation, non-receptive relaxation contributes to LES accommodation. Impaired receptive LES relaxation is the underlying cause of idiopathic EGJOO and achalasia. Besides the IRP value, the evaluation of two mechanisms, LES accommodation and swallow-induced LES relaxation is helpful for determining whether the LES relaxation function is impaired in clinical practice.

## Methods

### Patients

**Figure 5:**
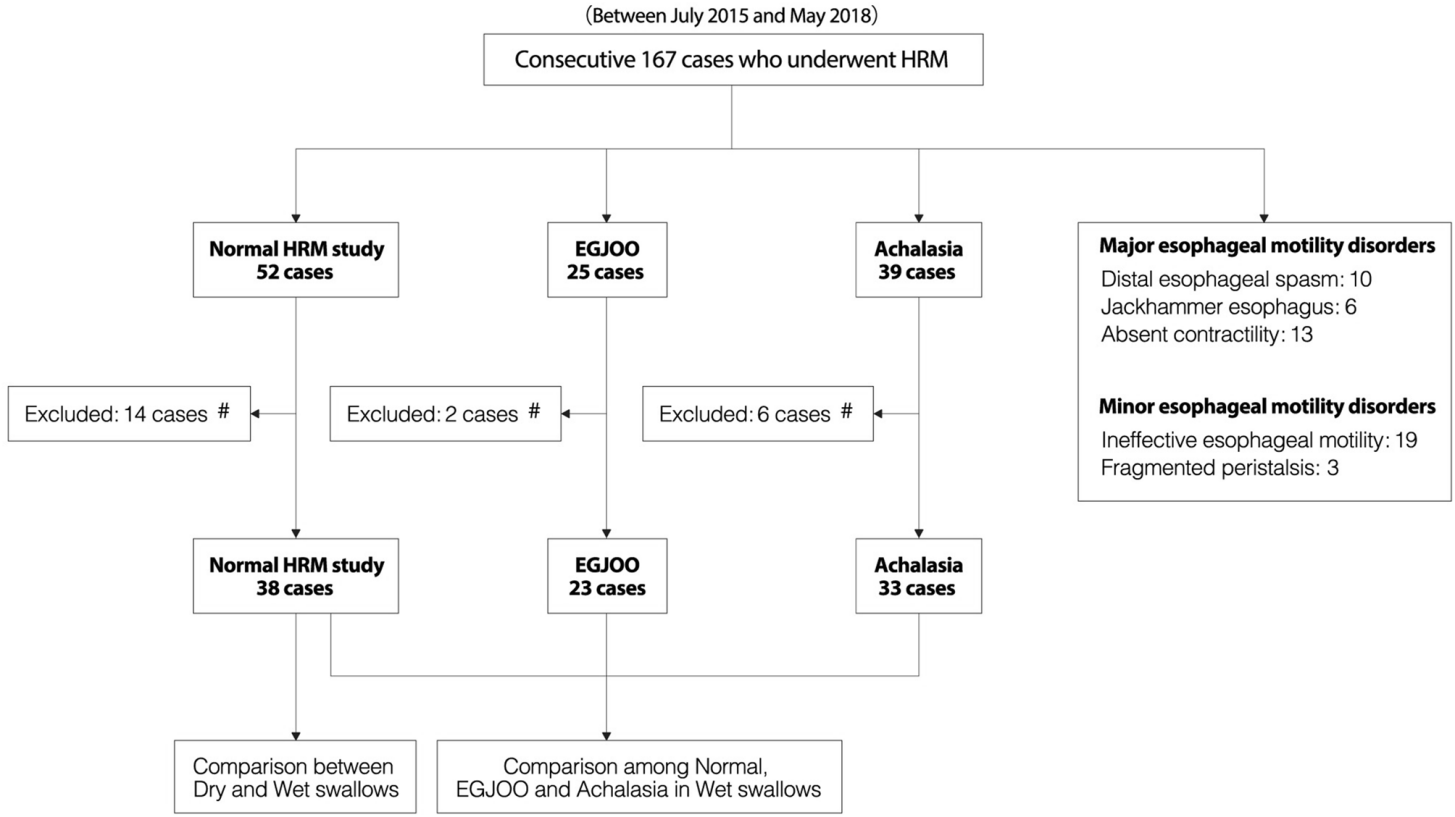
A flow chart showing enrollment of the patients in the present study. #Unsatisfactory HRM results for evaluation of LES accommodation. *EGJOO* esophagogastric junction outflow obstruction; *HRM* high-resolution manometry; *LES* lower esophageal sphincter.

A flow chart of this study is shown in Fig. 5. This was a prospective observational study. One hundred and sixty-seven consecutive patients with suspected EMDs who had some symptoms of chest discomfort and dysphagia and underwent HRM between July 2015 and May 2018 were included in the present study. Based on CC ver3.0, out of a total of 167 patients, HRM findings were normal in 52 patients, with the following major esophageal motility disorders detected: achalasia (n = 39), EGJOO (n = 25), distal esophageal spasm (n = 10), jackhammer esophagus (n = 6), absent contractility (n = 13) and minor esophageal disorders, including ineffective esophageal motility (n = 19) and fragmented peristalsis (n = 3). Fourteen of the 52 cases with normal HRM findings and 2 of the 25 cases of EGJOO were excluded because the HRM results were not suitable for the evaluation of LES accommodation. As a result, 38 cases with normal HRM findings were eligible for this study and analyzed to clarify the mechanisms by which successful LES relaxation was achieved during a wet swallow. No patients with normal HRM enrolled in this study had significant dysphagia, which was defined as a dysphagia score of Eckardt ≥ 2. In contrast, 23 cases with idiopathic EGJOO were analyzed to explore LES relaxation which was impaired during a wet swallow. For comparison purposes, 33 achalasia patients suitable for the evaluation of LES accommodation were included in this study to examine swallow-induced LES relaxation, LES accommodation and the LES accommodation index as a comparator. All of the enrolled patients had no history of thoracoabdominal surgery, and esophagogastroduodenoscopy (EGD) and computed tomography (CT) demonstrated no signs of other organic diseases, which ruled out secondary EGJOO.

### HRM protocol and analyses

**Figure 6:**
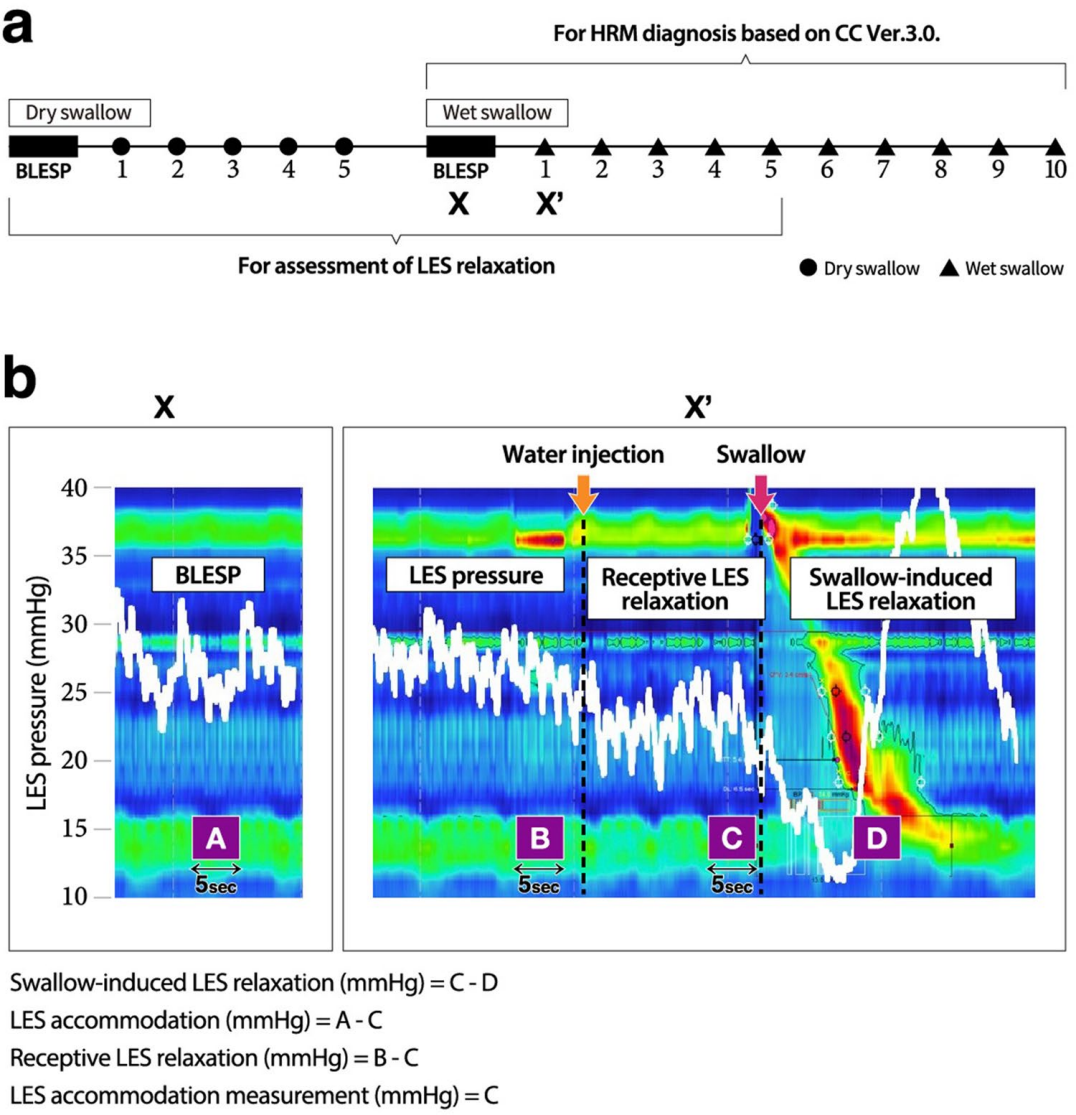
The protocol and original index of the present study. (**a**) The series of the study protocol is shown. After the basal lower esophageal sphincter pressure (BLESP) was obtained, the 5 dry swallows were performed. After the BLESP was obtained again, 10 wet swallows of 5 ml water were performed. (**b**) As for the original index in this study, swallow-induced LES relaxation, LES accommodation, receptive LES relaxation and LES accommodation measurement were determined using the following formulas: C–D, A–C, B–C and C, respectively. Black rectangles indicate the BLESP, and black circles and triangles indicate dry and wet swallows, respectively. A: The BLESP. B: The LES pressure for 5 s just before the injection of 5 mL of water into the oral cavity. C: The LES pressure during pharyngeal water stimulation for 5 s just before each swallow. D: The IRP level of each swallow. In this HRM image, the subject was asked to hold 5 mL water in the pharynx for 15 s longer than usual before swallowing, in order to make receptive LES relaxation easier to recognize.

The esophageal motility function was assessed by HRM using a ManoScan Z (Given Imaging, Los Angeles, CA, USA). HRM was performed using a standardized protocol with some minor modifications. After the basal condition without swallowing was recorded for 30 s, patients were instructed to swallow as infrequently as possible and to breathe quietly and regularly. At first, the patients were instructed to perform dry swallows 5 times at 1 min intervals in the supine position. Thereafter the patients were asked to perform 10 swallows of 5 ml water (wet swallow) at 1 min intervals in the supine position (Fig. 6a). In order to assess receptive LES relaxation (described later), the patients were instructed to hold 5 mL water in the pharynx for 5 s before swallowing. As the standard protocol, the diagnosis, based on CC ver3.0, was made using the 10 wet swallows. EGJOO is defined as EMD with impaired LES relaxation and abnormally high IRP (> 15 mmHg), but with normal or mildly abnormal peristalsis of the esophageal body. In order to assess the mechanisms of LES relaxation, we analyzed 5 dry swallows and the first 5 out of 10 wet swallows (Fig. 6a). We measured the HRM parameters, including the basal LES pressure (BLESP), integrated relaxation pressure (IRP), intrabolus pressure (IBP) at LES relaxation and IBP average max, distal contractile integral (DCI), and distal latency (DL), where BSLEP in this study was defined as the mean LES value for 5 s just before the 1st dry swallow or 1st wet swallow, whichever was applicable. In addition to the metrics mentioned above, an original index was used (Fig. 6b).

### Original index for the assessment of LES relaxation

In this study we assessed the mechanism of successful LES relaxation induced by a wet swallow with a focus on swallow-induced LES relaxation and the LES accommodation response, including receptive LES relaxation (Fig. 6b). Swallow-induced LES relaxation was defined as the mean value of 5 swallows obtained by subtracting IRP of each swallow from the mean value of the LES pressure for 5 s just before the swallow. LES accommodation in dry and wet swallows was defined as the mean value of 5 swallows obtained by subtracting the mean value of LES pressure for 5 s just before each swallow from the BLESP, which was obtained before the 1st dry and wet swallows, respectively. Receptive LES relaxation was defined as the mean value of 5 swallows obtained by subtracting the mean LES pressure for 5 s during pharyngeal water stimulation from that 5 s before injecting 5 mL water into the oral cavity. Considering the practical use of the evaluation for LES accommodation response, we proposed the LES accommodation measurement. The LES accommodation measurement is defined as the mean LES pressure of measurements performed 5 s before each of 10 wet swallows (Fig. 6b).

### Statistical analyses

All statistical analyses were performed using the Prism version 8.4.2. software program (GraphPad, San Diego, CA, USA). The data were expressed as the median (interquartile range). A paired or unpaired t-test was used to analyze continuous and ordinal data between two groups. A one-way ANOVA followed by Turkey’s multiple comparison test was conducted to analyze any differences among the three groups. A repeated measure analysis of variance followed by Dunnett’s test was used to determine statistical significance between more than two groups for the repeated data. Receiver operating characteristic (ROC) analyses were conducted to identify a more accurate cut-off point that could help differentiate EGJOO from normal HRM. ROC curves were created by plotting the range of sensitivity and specificity pairs for the diagnosis of EGJOO. *P* values of < 0.05 were considered to indicate statistical significance.

### Ethical considerations

The study protocol was approved by the Ethics Committee of Kyushu University, and was conducted in accordance with the ethical principles of the Declaration of Helsinki. All subjects provided their written informed consent before the start of the study.

## Abbreviations

BLESP: Basal LES pressure
CC: Chicago classification
DCI: Distal contractile integral
DL: Distal latency
EGJ: Esophagogastric junction
EGJOO: Esophagogastric junction outflow obstruction
EMD: Esophageal motility disorder
GERD: Gastroesophageal reflux disease
HRM: High-resolution manometry
IBP: Intrabolus pressure
IRP: Integrated relaxation pressure;
LES: Lower esophageal sphincter
LESR: Lower esophageal sphincter relaxation
PWS: Pharyngeal water stimulation
TLESR: Transient lower esophageal sphincter relaxation
